# Routine Molecular Surveillance of Drug-Resistant Tuberculosis: Translating Mutation Proxies into Clinical Governance Intelligence in Rural Eastern Cape

**DOI:** 10.3390/healthcare14101280

**Published:** 2026-05-08

**Authors:** Bulela Sonka, Mojisola Clara Hosu, Ntandazo Dlatu, Thokoe Vincent Makola, Lindiwe Modest Faye

**Affiliations:** 1 Walter Sisulu Tuberculosis Research Group, Faculty of Medicine and Health Sciences, School of Pathology, Walter Sisulu University, Mthatha 5100, South Africa; 222656956@mywsu.ac.za (B.S.); mhosu@wsu.ac.za (M.C.H.); 2 Walter Sisulu Institute for Clinical Governance and Healthcare Administration, Faculty of Medicine and Health Sciences, School of Public Health, Walter Sisulu University, Mthatha 5100, South Africa; ndlatu@wsu.ac.za (N.D.); tmakola@wsu.ac.za (T.V.M.)

**Keywords:** drug-resistant tuberculosis, molecular diagnostics, mutation proxies, clinical governance, surveillance, isoniazid resistance, fluoroquinolone resistance

## Abstract

Background: Drug-resistant tuberculosis (TB) remains a major challenge in high-burden settings, where timely identification of emerging resistance and effective governance responses are critical. While routine molecular diagnostics generate large volumes of resistance-associated mutation data, these outputs are typically used for individual patient management and remain underutilized for population-level surveillance and for the application of clinical governance approaches for improved TB care. Methods: We conducted a retrospective cross-sectional analysis of 1386 molecular diagnostic records for *Mycobacterium tuberculosis*, collected between March 2021 and December 2024, from 30 health facilities in the King Sabata Dalindyebo (K.S.D.) Local Municipality of Oliver Reginald (O.R.) Tambo District. Resistance-associated mutation proxies were identified for loci associated with isoniazid (*katG*, *inhA*), fluoroquinolone (*gyrA*), and second-line injectable agents (amikacin, kanamycin, and capreomycin) through mutations in the *rrs* locus. Mutation proxy prevalence was examined overall, by age group, over time, and across facilities. Persistence of resistance detection was assessed using consecutive-month analyses to characterize temporal continuity at the facility level. Results: At least one resistance-associated mutation proxy was detected in 72.7% of the analyzed records. Isoniazid resistance predominated, with *katG* mutation proxies identified in 52.2% and *inhA* in 20.2% of cases. Mutation proxies associated with fluoroquinolone and second-line injectable resistance were less frequently observed. Temporal analysis demonstrated variability over the study period, with a general decline in overall mutation proxy prevalence alongside a relative increase in *inhA*-associated mutations. Substantial heterogeneity in resistance patterns was observed across health facilities, with high-volume sites contributing the greatest absolute burden and selected facilities demonstrating sustained persistence of mutation detection over consecutive months. These findings highlight the magnitude, distribution, and persistence of resistance-associated mutation proxies within routine programmatic data. Conclusions: Routine molecular diagnostic data revealed a substantial and heterogeneous burden of drug-resistant *Mycobacterium tuberculosis* in K.S.D. Local Municipality, characterized by age-specific patterns, temporal shifts, and sustained facility-level persistence. Beyond descriptive epidemiology, routinely generated mutation proxy data can serve as early-warning indicators of clinical governance stress, signaling emerging pressures on TB care systems when resistance patterns persist or worsen. Interpreting these trends can support more anticipatory clinical governance, strengthen resistance surveillance, and guide prioritized interventions in high-burden, resource-constrained settings.

## 1. Introduction

Tuberculosis (TB) remains a leading cause of morbidity and mortality globally, with drug-resistant forms posing a persistent threat to TB control efforts [1,2]. South Africa is among the countries with the highest burden of both drug-sensitive and drug-resistant TB, where resistance to first-line and second-line anti-TB drugs complicates treatment and undermines programmatic outcomes [3,4]. Understanding the distribution and patterns of drug resistance is therefore critical for informing clinical management at the individual patient level and public health response strategies at the health system level.

The widespread implementation of molecular diagnostic assays has transformed TB diagnosis by enabling rapid detection of *Mycobacterium tuberculosis* and key resistance-associated mutations [5,6]. These assays generate routine data on resistance-associated genetic changes at loci such as *katG*, *inhA*, *gyrA*, and *rrs*, which are commonly used as proxies for resistance to isoniazid, fluoroquinolones, and injectable agents. While such mutation proxy data are primarily used to guide individual patient management, they also represent an underutilized resource for describing population-level patterns of drug resistance and for informing policy and practice guidelines.

In South Africa, routine TB diagnosis and resistance detection are primarily conducted using molecular diagnostic assays, including GeneXpert MTB/RIF as the initial rapid test for detection of *Mycobacterium tuberculosis* and rifampicin resistance. Additional resistance profiling is performed using Xpert MTB/XDR and line probe assays (LPAs), which provide mutation-level information for isoniazid (*katG*, *inhA*), fluoroquinolones (*gyrA*), and second-line injectable agents (*rrs*, *eis*). These platforms generate routinely available mutation proxy data that extend beyond rifampicin resistance and offer opportunities for more detailed surveillance of evolving resistance patterns in programmatic settings. Although rifampicin resistance is a key marker of multidrug-resistant tuberculosis, this study focuses on additional resistance-associated loci to leverage the underutilized potential of routinely generated mutation proxy data beyond rifampicin resistance. This approach enables more refined characterization of evolving resistance patterns, particularly for isoniazid and second-line drugs, and supports anticipatory, data-driven clinical governance strategies.

Most epidemiological studies of drug-resistant TB rely on culture-based drug susceptibility testing or whole-genome sequencing, approaches that may be limited by cost, turnaround time, and incomplete coverage in resource-constrained settings [7]. In contrast, routinely generated molecular diagnostic data are widely available and reflect resistance patterns encountered in everyday clinical practice. However, these data are often under-analysed beyond aggregate reporting, and few studies have systematically examined variation in resistance-associated mutation proxies by age, time, and health facility.

In high-burden districts such as Oliver Reginald (O.R.) in the Eastern Cape Province, healthcare access, referral pathways, and patient populations vary substantially across facilities [8]. As a result, aggregated resistance estimates may obscure important heterogeneity patterns within the burden of drug-resistant TB. Disaggregated analyses that consider temporal trends, age-specific patterns, and facility-level variation are therefore essential for a more nuanced understanding of resistance dynamics.

Beyond descriptive epidemiology, these findings highlight the potential to repurpose routinely generated mutation proxy data as early-warning indicators of clinical governance stress, which are situations in which healthcare systems or facilities experience strain in maintaining the quality, safety, and effectiveness of clinical care due to emerging risks, increasing service pressure, or gaps in oversight and response mechanisms. Such stress typically becomes evident when routine clinical or diagnostic indicators begin to signal persistent or worsening disease patterns that require coordinated clinical, managerial, and programmatic responses. Interpreting mutation proxy trends through this governance lens can support more anticipatory clinical governance, strengthen oversight of TB-resistance diagnostics, and enable prioritized interventions in high-burden, resource-constrained settings.

In this study, routinely generated data from GeneXpert MTB/RIF, Xpert MTB/RIF Ultra, and line probe assays (LPAs) were used to derive resistance-associated mutation proxies. The study aimed to describe the prevalence and distribution of resistance-associated mutation proxies in *Mycobacterium tuberculosis* using routine molecular diagnostic data collected over 4 years in the King Sabata Dalindyebo (K.S.D.) Local Municipality in the O.R. Tambo District, Eastern Cape, South Africa. Specifically, we examined overall and gene-specific mutation proxy prevalence, temporal variation, age-stratified patterns, facility-level heterogeneity, and the persistence of resistance detection over time. By leveraging routinely collected diagnostic data, this study provides real-world insights into patterns of drug-resistant TB in a high-burden setting.

## 2. Materials and Methods

### 2.1. Study Design and Data Source

This study employed a retrospective cross-sectional design using secondary laboratory data derived from routinely generated molecular TB diagnostic tests. The data were collected from health facilities within the King Sabata Dalindyebo Local Municipality (KSDLM), O.R. Tambo District, Eastern Cape, South Africa, between March 2021 and December 2024. The dataset comprised molecular diagnostic records generated as part of routine TB screening and drug-resistance testing conducted within standard clinical care pathways. These diagnostic procedures were implemented programmatically across participating health facilities, rather than for research purposes, and therefore reflect real-world diagnostic practices and resistance patterns encountered in routine clinical settings. The unit of analysis was individual diagnostic test records rather than unique patients, as the anonymized dataset lacked consistent patient identifiers across facilities. All *Mycobacterium tuberculosis*-positive molecular diagnostic records generated during the study period were eligible for inclusion. Records with missing key variables, including mutation proxy results or facility identifiers, were excluded from relevant analyses.

Molecular diagnostic data were generated using WHO-endorsed rapid molecular assays routinely deployed within the South African TB program, specifically the GeneXpert MTB/RIF and Xpert MTB/RIF Ultra platforms. These cartridge-based nucleic acid amplification tests detect *Mycobacterium tuberculosis* complex DNA and rifampicin resistance by targeting mutations within the rifampicin-resistance-determining region (RRDR) of the rpoB gene through probe-based hybridization signals. The dataset included detailed probe-level outputs, which were used as proxies for resistance-associated mutations. Where available through programmatic workflows, additional mutation proxies (e.g., *katG* and *inhA*) were incorporated from extended diagnostic records. These data represent routine clinical testing outputs rather than comprehensive molecular sequencing.

### 2.2. Study Setting and Population

**Figure 1:**
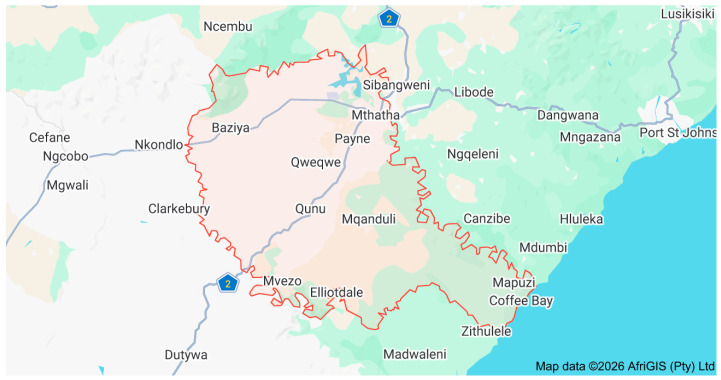
Map of the King Sabata Dalindyebo Local Municipality (KSDLM), showing its 35 wards and the seat of Mthatha. (Source: Adapted from King Sabata Dalindyebo Local Municipality [9].

The study was conducted in KSDLM, a category B municipality in the O.R. Tambo District, Eastern Cape Province, South Africa. The municipality covers approximately 3027 km^2^ and comprises 37 wards, accounting for a substantial proportion of the district’s geographic area (Figure 1).

KSDLM has an estimated population of approximately 476,558, 114,580 of whom reside in households, with around 95% living in rural areas. The population is predominantly African, with IsiXhosa as the primary language. The area is characterized by high burdens of TB and HIV, limited healthcare access in rural settlements, and persistent socioeconomic inequalities that influence health-seeking behavior and access to care. The analysis included 1386 molecular diagnostic test records from 30 health facilities within the municipality. Available variables included test date, facility identifier, patient age, and assay-derived probe outputs for resistance-associated mutation proxies. Gender data were not available in the dataset and were therefore not included in the analysis.

#### Definition of Resistance-Associated Mutation Proxies

Molecular diagnostic data used in this study were generated through routinely implemented WHO-endorsed rapid molecular assays within the South African tuberculosis control program. These included GeneXpert MTB/RIF and Xpert MTB/RIF Ultra platforms, which are cartridge-based nucleic acid amplification tests designed to detect *Mycobacterium tuberculosis* complex DNA and rifampicin resistance through probe-based hybridization targeting the *rpoB* gene. In addition, LPAs were used, where available, for extended resistance profiling, enabling detection of mutations in genes associated with isoniazid (*katG*, *inhA*), fluoroquinolones (*gyrA*), and second-line injectable agents (*rrs*, *eis*). These molecular assays operate by amplifying specific genomic regions of *Mycobacterium tuberculosis* and detecting mutations through changes in probe binding or melt peak temperature signals. The outputs are interpreted as genotypic indicators of resistance-associated genetic regions. In this study, these assay-derived signals were used as mutation proxies to characterize resistance patterns at the population level. The analysis was restricted to resistance-associated loci detectable through routinely deployed molecular diagnostic platforms, including *katG*, *inhA*, *gyrA*, and *rrs*, and does not represent comprehensive genomic resistance profiling.

Rifampicin-resistance mutation data were not included in this analysis. Although rifampicin resistance is routinely detected using GeneXpert MTB/RIF within standard diagnostic algorithms and serves as a key marker of multidrug-resistant tuberculosis, the present study intentionally focuses on additional resistance-associated loci. This approach leverages the underutilized potential of routinely generated mutation proxy data beyond rifampicin resistance, enabling more granular characterization of emerging resistance patterns, particularly for isoniazid and second-line drugs. By doing so, the analysis provides complementary insights that support earlier detection of resistance evolution and inform more anticipatory, data-driven clinical governance responses in high-burden settings. Resistance-associated mutation proxies were defined using mutant melt peak temperature signals generated by WHO-endorsed molecular diagnostic assays [10,11].

These include GeneXpert MTB/RIF for the detection of *Mycobacterium tuberculosis* and rifampicin resistance, and LPAs for extended resistance profiling. These assays produce probe- or melt-peak-based signals corresponding to resistance-associated genetic regions. LPAs provide mutation-level information for isoniazid (*katG*, *inhA*), fluoroquinolones (*gyrA*), and second-line injectable agents (*rrs*, *eis*), based on probe hybridization or melt peak temperature shifts associated with specific resistance-conferring genetic regions. In this study, a mutation proxy was considered present when a mutant probe signal or melt peak was detected at a resistance-associated locus, and absent when no such signal was observed. These assay-derived outputs represent molecular indicators of resistance-associated genetic variation rather than sequencing-confirmed mutations and are routinely generated as part of standard TB diagnostic workflows.

A mutation proxy was classified as present when a mutant melt peak was detected for a resistance-associated locus and absent when no such signal was observed. Proxies were defined for *katG* and *inhA* as indicators of isoniazid resistance, *gyrA* (any region) as an indicator of fluoroquinolone resistance, *rrs* as an indicator of resistance to injectable agents, and *eis* as an indicator of kanamycin resistance. In line with the WHO SMART guideline terminology [12,13], molecular assays are designed to detect “mutations associated with resistance to anti-tuberculosis medicines.” They are intended to support “routine surveillance of drug resistance using data generated through diagnostic services.” Accordingly, these mutation proxies are interpreted as assay-derived molecular indicators rather than sequencing-confirmed mutations. Their use is appropriate for population-level surveillance and clinical governance of TB management, as they are generated routinely during standard care, enable timely identification of emerging resistance patterns, and provide actionable intelligence to inform facility-level monitoring, prioritization, and early clinical governance intervention to improve clinical outcomes in high-burden settings. These routinely generated assay-derived molecular indicators provide a practical and scalable data source for population-level surveillance and clinical governance in high-burden settings. The mutation proxies represent assay-derived molecular indicators of resistance-associated genetic regions and should not be interpreted as sequencing-confirmed mutations. Additional analyses were conducted to assess temporal persistence of mutation proxies (defined as detection over ≥6 consecutive months) and facility-level clustering to identify localized patterns of resistance burden.

### 2.3. Molecular Diagnostic Methods

Molecular diagnostic data used in this study were generated through routinely implemented WHO-endorsed rapid molecular assays within the South African tuberculosis control program. These included GeneXpert MTB/RIF and Xpert MTB/RIF Ultra platforms as primary diagnostic tools, with additional resistance profiling performed using LPAs.

These assays amplify specific DNA sequences of *Mycobacterium tuberculosis* and detect mutations via probe-based hybridization within defined genomic regions. Rifampicin resistance is identified by mutations in the RRDR of the *rpoB* gene, as evidenced by changes in probe binding patterns.

Where available through extended diagnostic workflows, additional mutation proxies were included, such as *katG* and *inhA* (associated with isoniazid resistance) and *gyrA* (associated with fluoroquinolone resistance).

These mutation proxies were derived from assay-generated probe outputs and interpreted as indicators of genotypic resistance. It is important to note that these assays detect only predefined, clinically relevant mutations and do not provide comprehensive genomic sequencing or full resistance profiling. The data, therefore, represent genotypic resistance patterns identified through routine diagnostic testing, rather than phenotypically confirmed resistance.

While these molecular assays provide rapid and programmatically valuable detection of resistance-associated genetic regions, they may not fully correspond to phenotypic drug susceptibility testing, and some level of discordance is recognized, particularly for mutations associated with low-level or borderline resistance.

### 2.4. Outcomes and Stratification

The unit of analysis in this study was individual test records rather than unique patients, as anonymized laboratory data did not include consistent patient identifiers across all facilities. Where possible, records were screened for exact duplicates based on identical facility, date, and assay outputs, and such duplicate entries were removed. However, repeat tests from the same individual could not be fully excluded and were retained as part of routine programmatic data. Records with missing key variables (e.g., mutation proxy results or facility identifiers) were excluded from relevant analyses, while variables with partial missingness were analyzed using available-case approaches.

The primary outcome was the prevalence of resistance-associated mutation proxies, defined as the proportion of tests with at least one detected proxy. Secondary analyses examined gene-specific prevalence, monthly and annual variation, age-stratified distribution, facility-level variation, and persistence of mutation detection. Age was categorized into standard epidemiological groups: <15, 15–24, 25–34, 35–44, 45–54, and ≥55 years.

### 2.5. Temporal and Facility-Level Analyses

Monthly and annual prevalence estimates were calculated using the test date. Facility-level analyses were restricted to facilities with ≥10 tests to ensure stable estimates. Facilities were assigned anonymized codes for reporting. Persistence of resistance detection was assessed by identifying consecutive months with at least one mutation-positive test at each facility. A separate analysis was conducted for *gyrA* mutation proxies.

### 2.6. Statistical Analysis

Data were analyzed using Microsoft Excel 365 (version 16.x; Microsoft Corporation, Redmond, WA, USA) for data cleaning and preliminary summaries. Descriptive statistical analyses, temporal aggregation, and data visualization were conducted using Python (version 3.10; Python Software Foundation, Wilmington, DE, USA), with the pandas library (version 2.1; USA) for data manipulation, NumPy (version 1.26; Wilmington, DE, USA) for numerical operations, and matplotlib (version 3.8; Wilmington, DE, USA) for graphical visualization. The analysis was primarily descriptive, focusing on frequencies, proportions, and temporal patterns. No causal inference was intended. Where applicable, trends were interpreted cautiously, given the observational nature of the data. Data inclusion criteria, variable definitions, and analytical procedures were predefined and applied consistently across all facilities to ensure comparability and reproducibility of results. The analytical logic of this study is grounded in the use of routinely generated molecular diagnostic data to explore patterns of drug-resistance-associated mutations within a real-world clinical setting. Rather than aiming to develop predictive or causal models, the study adopts an exploratory and descriptive approach to examine how resistance patterns are distributed across programmatic data. The scientific contribution lies in demonstrating how routinely collected molecular data can be leveraged to generate context-specific insights into resistance dynamics, particularly in resource-limited settings where structured epidemiological datasets are often unavailable. By focusing on mutation proxy patterns and their distribution, the study provides a practical, data-driven perspective on drug-resistant TB that complements existing molecular epidemiology research and highlights the potential of routine diagnostic systems as sources of epidemiologically relevant information.

### 2.7. Ethical Considerations

The study utilized de-identified secondary laboratory data generated through routine diagnostic services. Ethical clearance was obtained from the Research Ethics and Biosafety Committee, Faculty of Health Sciences, Walter Sisulu University (Ref: WSU HREC 141/2025; approved 2 July 2025). The Eastern Cape Department of Health granted administrative permission (Ref: EC_202507_023; approved 11 July 2025). National Health Laboratory Services granted the collection of laboratory diagnostic patient sample result data (Ref: SR4169693; approved 25 November 2025).

## 3. Results

These findings reflect assay-derived genotypic resistance patterns and should not be interpreted as direct measures of phenotypic drug resistance, given known discordance between molecular and phenotypic testing approaches.

The results are presented as a structured descriptive analysis of routinely generated molecular diagnostic data, with emphasis on identifying patterns, distributions, and temporal and facility-level variation in resistance-associated mutation proxies. Given the nature of the dataset and study design, the analysis does not focus on inferential statistical testing but rather on characterizing epidemiologically relevant trends within real-world programmatic data. To enhance clarity and minimize redundancy, tables and figures were selected to provide complementary, non-overlapping representations of key findings. Overall, the analysis indicates that resistance-associated mutation proxies are widely distributed across the dataset, with isoniazid resistance predominating and clear evidence of temporal variation and facility-level heterogeneity. These observed patterns may reflect the influence of transmission dynamics, treatment-related selective pressure, and variations in service delivery contexts. However, given the reliance on molecular diagnostic proxies, the findings should be interpreted cautiously as indicators of genotypic resistance patterns, rather than phenotypically confirmed resistance.

In addition to overall prevalence, patterns of mutation persistence were observed, with certain resistance-associated mutation proxies detected consistently over consecutive months (≥6 months) within specific facilities. This sustained detection suggests ongoing transmission or programmatic gaps in treatment response and follow-up. Furthermore, facility-level clustering was evident, with a small number of high-volume facilities contributing disproportionately to the overall burden of detected mutation proxies. These findings highlight non-random distribution patterns within the dataset, indicating localized concentration of resistance and potential hotspots requiring targeted intervention.

### 3.1. Diagnostic Volume and Dataset Characteristics

A total of 1386 molecular diagnostic records were analyzed, representing tests conducted between March 2021 and December 2024 across 30 health facilities within KSDLM. Diagnostic volume increased markedly over time, with most tests conducted in 2024 (70.4%), followed by 2023 (22.2%), 2022 (5.3%), and 2021 (2.1%). This progressive increase likely reflects expanded access to molecular diagnostics and scaling of TB testing services, rather than a direct increase in disease burden. Consequently, temporal comparisons of mutation prevalence should be interpreted in the context of changing diagnostic coverage and testing intensity.

### 3.2. Prevalence and Distribution of Resistance-Associated Mutation Proxies

Overall, 1007 of 1386 tests (72.7%) showed at least one resistance-associated mutation proxy. These proxies represent assay-derived indicators of resistance-associated genomic regions, rather than sequencing-confirmed mutations. Isoniazid-resistance-associated mutation proxies predominated, with *katG* detected in 52.2% and *inhA* in 20.2% of records. This pattern is consistent with known mechanisms of isoniazid resistance, in which *katG* mutations are commonly associated with high-level resistance, and *inhA* mutations with low-level resistance and potential cross-resistance to related drugs. In contrast, mutation proxies associated with second-line drugs, including fluoroquinolones and injectable agents, were observed less frequently, suggesting either lower prevalence of second-line resistance or limitations in routine diagnostic detection capacity.

### 3.3. Temporal Patterns in Drug-Resistance Mutation Proxies

#### 3.3.1. Monthly Variation

**Figure 2:**
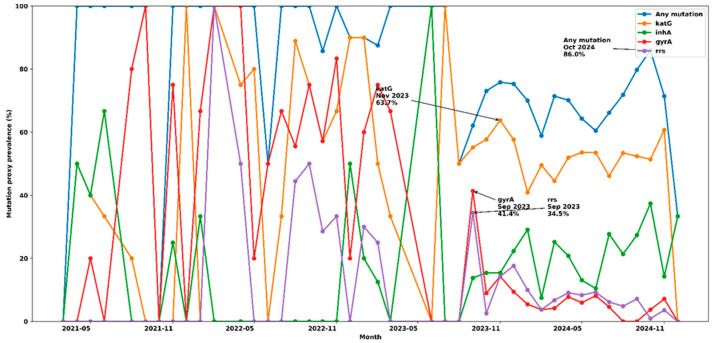
Monthly variation in resistance-associated mutation proxy prevalence.

Figure 2 below demonstrates marked month-to-month variability in resistance-associated mutation proxy prevalence, against a consistently high background prevalence of mutation proxies detected by molecular diagnostic tests. The prevalence of any mutation proxy remained elevated throughout the study period, peaking in October 2024, when 86.0% (92/107) of tests were mutation-positive, indicating sustained high levels of possible drug-resistant *Mycobacterium tuberculosis*. Isoniazid resistance, reflected by *katG* mutation proxies, showed persistent but fluctuating prevalence, with a prominent peak in November 2023 (63.7%; 58/91), suggesting episodic intensification rather than a uniform temporal trend. In contrast, second-line resistance proxies displayed more episodic behaviour, with sharp, temporally clustered peaks in September 2023 for both *gyrA* (41.4%; 12/29) and *rrs* (34.5%; 10/29), indicating periods of increased presentation or detection of more complex resistance patterns. The *inhA* mutation proxies remained lower overall but increased later in the study period, consistent with a shifting resistance profile over time (Figure 2). Overall, the figure highlights a pattern of sustained baseline resistance punctuated by discrete temporal surges, particularly for second-line resistance markers, underscoring the importance of longitudinal monitoring, as short-term peaks may signal emerging resistance pressure or delayed clinical and programmatic responses that require timely review.

#### 3.3.2. Annual Variation

Annual aggregation demonstrated clear temporal shifts in the prevalence of resistance-associated mutation proxies, as indicated (Table 1). The prevalence of any mutation proxy declined progressively from 95.9% (70/73) in 2022 to 74.7% (230/308) in 2023, and then to 70.0% (684/976) in 2024. Gene-specific trends showed divergent patterns over time. The prevalence of *katG* mutation proxies decreased steadily from 68.5% in 2022 to 59.7% in 2023 and 49.5% in 2024, while *inhA* mutation proxies increased from 8.2% to 17.2% and further to 22.0% over the same period. In contrast, second-line resistance proxies declined markedly, with *gyrA* mutation prevalence falling from 57.5% in 2022 to 17.5% in 2023 and 4.3% in 2024, and *rrs* mutation proxies decreasing from 24.7% to 14.6% and 6.4%, respectively. These temporal trends should be interpreted in the context of evolving DR-TB treatment practices during the study period, including increased adoption of all-oral regimens and reduced reliance on second-line injectable agents, which may influence selective drug pressure and the observed distribution of resistance-associated mutation proxies.

##### Annual Variation in Resistance-Associated Mutation Proxy Prevalence

**Figure 3:**
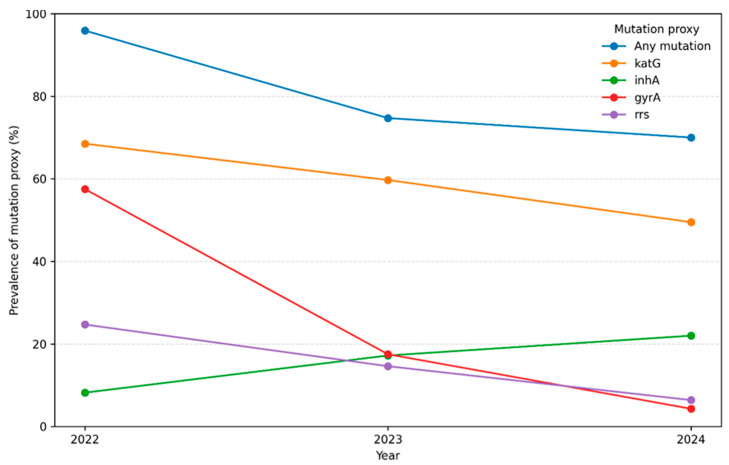
Annual prevalence of resistance-associated mutation proxies between 2022 and 2024. Lines represent the proportion of molecular diagnostic tests with detected mutation proxies for any resistance, *katG*, *inhA*, *gyrA*, and *rrs.*

Figure 3 below illustrates changes in the annual prevalence of resistance-associated mutation proxies between 2022 and 2024. The prevalence of any mutation proxy declined over the study period, decreasing from 95.9% in 2022 to 74.7% in 2023 and 70.0% in 2024. Gene-specific trends varied across resistance loci. The prevalence of *katG* mutation proxies decreased progressively from 68.5% in 2022 to 59.7% in 2023 and 49.5% in 2024. In contrast, *inhA* mutation proxies increased over time, rising from 8.2% in 2022 to 17.2% in 2023 and 22.0% in 2024. For second-line resistance proxies, the prevalence of *gyrA* mutations declined markedly from 57.5% in 2022 to 17.5% in 2023 and 4.3% in 2024, while *rrs* mutation proxies decreased from 24.7% to 14.6% and 6.4% over the same period.

### 3.4. Age-Stratified Distribution of Drug-Resistance Mutation Proxies

Resistance-associated mutation proxies were detected across all adult age groups (Table 2). The 15–24-year age group demonstrated the highest prevalence of any mutation proxy, with 153 of 157 tests (97.5%) testing positive, predominantly due to *katG* mutations. Estimates for the <15-year age group should be interpreted with caution due to the small sample size, which may result in unstable percentage estimates. The 25–34-year and 35–44-year age groups contributed the largest absolute numbers of mutation-positive tests and showed the greatest diversity of resistance-associated mutation proxies, including substantial detection of *gyrA* and *rrs* mutations.

### 3.5. Facility-Level Distribution of Drug-Resistance Mutation Proxies

**Figure 4:**
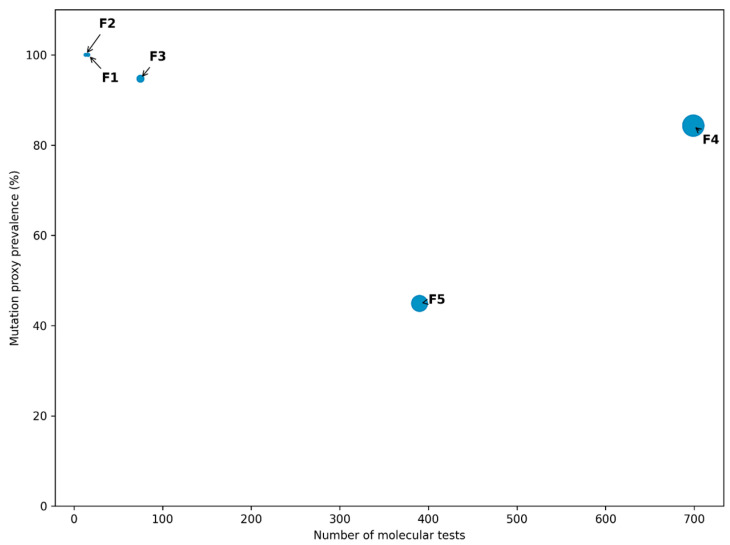
Facility-level distribution of resistance-associated mutation proxy prevalence and diagnostic volume.

Facility (F)-level analysis (Figure 4) revealed marked heterogeneity in the prevalence and distribution of resistance-associated mutation proxies across health facilities. Several sites exhibited extremely high mutation proxy prevalence, including F1 (16/16; 100%), F2 (13/13; 100%), and F3 (71/75; 94.7%), although these estimates were influenced by small diagnostic volumes at some facilities. In contrast, F4 contributed the largest absolute number of mutation-positive tests (589/699; 84.3%), reflecting its substantially higher diagnostic throughput rather than disproportionately elevated prevalence alone. When prevalence and diagnostic volume were considered jointly, substantial inter-facility heterogeneity became evident, with some facilities characterized by very high-percentage positivity but low testing volume, and others contributing a high absolute burden of resistant cases due to large service volumes. Facility-level estimates should be interpreted alongside diagnostic volume, as facilities with low testing numbers may yield unstable percentage estimates that do not reflect broader population-level patterns.

### 3.6. Persistence of Drug-Resistance Detection over Time

**Figure 5:**
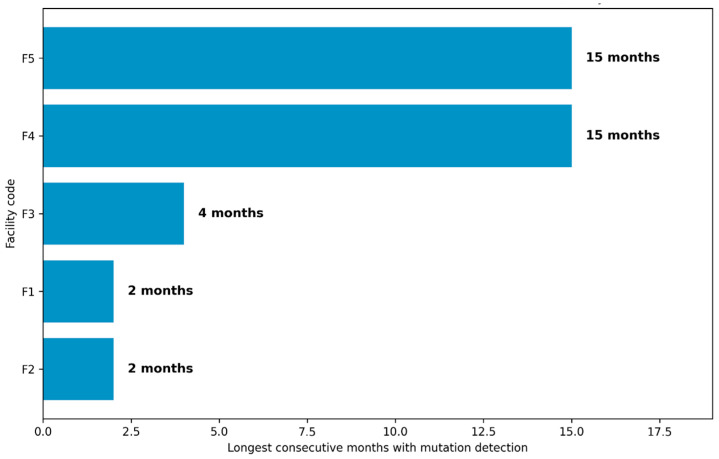
Duration of the longest uninterrupted period of resistance-associated mutation detection at selected health facilities.

Consecutive months with mutation-positive tests were observed at selected facilities (Figure 5). F4 demonstrated mutation-positive results in 29 of 46 months, with a longest continuous run of 15 months. F2 demonstrated mutation-positive results in 19 of 36 months, also with a longest continuous run of 15 months. Other facilities showed shorter continuous runs of mutation detection, ranging from 2 to 5 months. Analysis restricted to fluoroquinolone-associated *gyrA* mutation proxies showed prolonged recurrence at F4, with *gyrA* mutations detected in 27 months and uninterrupted runs of up to 14 months.

**Figure 6:**
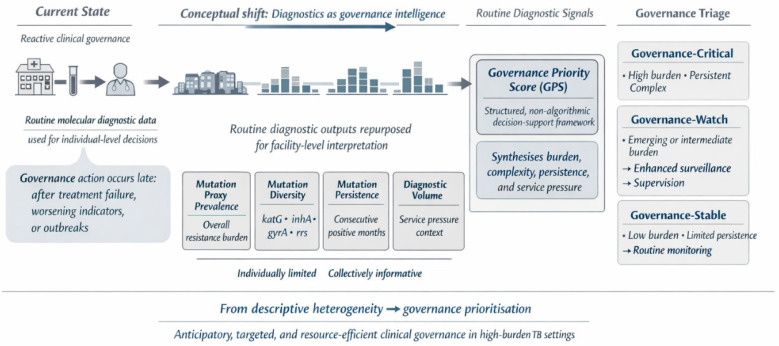
Facility-level governance triage model based on routine molecular diagnostic data.

The Facility-Level Governance Priority Score (GPS) conceptual model (Figure 6) integrates four routinely available indicators: resistance-associated mutation proxy prevalence; mutation diversity across *katG*, *inhA*, *gyrA*, and *rrs* loci; persistence of mutation detection over consecutive months; and diagnostic volume to stratify facilities into governance-critical, governance-watch, and governance-stable categories. This framework reframes facility-level heterogeneity from a descriptive epidemiological finding into a decision-support tool for prioritizing anticipatory clinical governance interventions to enhance the response to emerging patterns of drug resistance. Clinical governance, in this instance, would entail analyzing and monitoring resistance patterns against clinical outcome trends to determine the clinical significance of these mutation proxy trends for current TB management approaches.

Application of the GPS framework to selected high-impact facilities demonstrated clear differentiation in governance risk profiles (Table 3). Facilities F2 and F4 were classified as governance-critical, reflecting the convergence of resistance complexity, including substantial second-line resistance, and, in the case of F4, high diagnostic volume with prolonged persistence of mutation detection. In contrast, facilities F1, F3, and F5 were classified as governance-watch facilities, characterized by high proportional resistance prevalence or notable second-line signals but lower diagnostic volume, a referral-driven case mix, or limited evidence of sustained persistence. No facilities within this subset met the criteria for governance-stable classification.

## 4. Discussion

This study moves beyond descriptive reporting by identifying programmatically meaningful patterns in routinely generated molecular diagnostic data. The high prevalence of resistance-associated mutation proxies (72.7%), together with the predominance of *katG* and *inhA* mutations, indicates a substantial burden of isoniazid resistance within the study setting. Importantly, the detection of mutation persistence over extended periods (≥6 months) suggests a potential early warning signal of ongoing transmission, delayed treatment response, or gaps in adherence and follow-up. In addition, the observed facility-level clustering demonstrates that resistance burden is not uniformly distributed but concentrated within specific service points. Collectively, these findings highlight the value of routine molecular data as a tool for clinical governance, enabling targeted intervention planning and strengthening surveillance intelligence in high-burden settings.

While the findings are descriptive, they provide important insights into the distribution and potential drivers of drug-resistant tuberculosis within the study setting. The predominance of *katG*-associated mutation proxies is consistent with well-established mechanisms of high-level isoniazid resistance and may reflect both historical treatment exposure and ongoing transmission of resistant strains. The observed increase in *inhA*-associated mutations over time may indicate shifting resistance dynamics, potentially linked to treatment practices or selective drug pressure. The concentration of resistance burden among individuals aged 25–44 years suggests a possible intersection between active transmission, social mobility, and HIV co-infection, which is known to be prevalent in this age group in high-burden settings. Facility-level heterogeneity further indicates that resistance patterns are not uniformly distributed, but may reflect localized differences in diagnostic access, patient referral pathways, or treatment program performance. The persistence of mutation detection over extended periods at specific facilities may indicate sustained transmission chains or challenges with treatment effectiveness and adherence. While causal inference cannot be established, these patterns highlight areas where enhanced surveillance, targeted interventions, and improved treatment monitoring may be warranted.

It is important to note that molecular resistance markers may not fully align with phenotypic drug susceptibility testing results, and some discordance has been reported in the literature; however, the use of routinely generated molecular data remains appropriate for surveillance and programmatic monitoring in high-burden settings.

### 4.1. Diagnostic Volume and Dataset Maturity

Our study analyzed 1386 molecular diagnostic records collected between March 2021 and December 2024 across 30 health facilities in KSDLM, in the O.R. Tambo District municipality of the Eastern Cape, South Africa. Diagnostic volume increased markedly over time, with more than 70% of tests performed in 2024, reflecting the progressive expansion and routine integration of molecular diagnostics into district TB services. Earlier years contributed smaller sample sizes and likely reflect a more selective testing phase focused on higher-risk patients. This pattern may also have been influenced by disruptions to routine health services during the COVID-19 pandemic, which affected TB case detection, diagnostic testing volumes, and healthcare access in many high-burden settings [14,15,16]. Nevertheless, the multi-year span of the dataset provides a valuable longitudinal perspective, enabling assessment of evolving drug-resistance patterns as diagnostic coverage matured within routine clinical practice.

### 4.2. Burden of Drug-Resistance Mutation Proxies

The predominance of *katG*-associated mutation proxies in this study is consistent with global and regional evidence showing that mutations in *katG*, particularly codon 315, are the dominant mechanism of isoniazid resistance in *Mycobacterium tuberculosis* [10,17]. These findings should be interpreted in the context of assay-derived mutation proxies, which reflect molecular signals detected by diagnostic platforms rather than sequencing-confirmed resistance mutations. African molecular epidemiology studies have similarly reported persistent transmission and evolution of drug-resistant TB strains, with resistance dynamics influenced by treatment pressures and population-level factors such as HIV co-infection [18,19]. These findings suggest the value of routinely generated molecular diagnostic data as surveillance tools, enabling early detection of emerging resistance signals and supporting anticipatory clinical governance and strengthened programmatic TB-resistance monitoring in high-burden settings. The concurrent detection of *inhA* mutation proxies (20.2%) in this study indicates the coexistence of multiple mechanisms of isoniazid resistance within the same population. This pattern is consistent with findings from several African settings where *katG* mutations, typically associated with high-level isoniazid resistance, coexist with *inhA* promoter mutations that confer lower-level resistance through target overexpression [20]. Similar distributions of *katG* and *inhA* mutations have been reported in studies from Cameroon and other African regions, highlighting the heterogeneous molecular landscape of isoniazid resistance across the continent [20,21]. Research conducted in the Eastern Cape has further demonstrated that undetected isoniazid monoresistance, particularly linked to *inhA* promoter mutations, represents a critical pathway for the emergence of multidrug-resistant tuberculosis, especially when routine diagnostic algorithms prioritize detection of rifampicin resistance [22]. Comparable mutation patterns have also been observed in studies from Uganda and Ghana, where *katG* S315T remains the dominant mutation, while *inhA* promoter mutations contribute substantially to resistance diversity [23,24]. In contrast, the relatively low detection of fluoroquinolone-associated (*gyrA*) and injectable-associated (*rrs*) mutation proxies, together with the absence of *eis* mutations, is consistent with the well-described stepwise accumulation of drug resistance in *Mycobacterium tuberculosis*, where resistance to first-line drugs typically precedes the emergence of resistance to second-line agents. This pattern reflects the prolonged historical use of first-line drugs such as isoniazid and rifampicin in TB treatment programs, which creates sustained selective pressure favoring mutations in genes such as *katG* and *inhA*. In contrast, mutations conferring resistance to second-line agents remain comparatively rare. Similar findings have been reported in studies from Ethiopia and other high-burden settings, where resistance to first-line drugs substantially exceeds resistance to fluoroquinolones or injectable agents [25]. In that study, resistance to any second-line drug was detected in only 5.6% of isolates, including a small proportion of pre-XDR and extensively drug-resistant (XDR) cases, highlighting the comparatively limited spread of second-line resistance despite widespread first-line resistance [25].

This gradient in resistance acquisition is also supported by epidemiological and modeling studies demonstrating that resistance typically emerges through sequential genetic adaptation during inadequate or interrupted treatment, with first-line resistance creating a pathway for the subsequent accumulation of additional mutations leading to multidrug-resistant (MDR) TB, pre-XDR-TB, and XDR-TB. Mathematical modeling frameworks further illustrate how the transition from drug-susceptible infection to first-line resistance, and eventually to second-line resistance, can occur through progressive selection under treatment pressure, reinforcing the concept that first-line resistance serves as the initial gateway in the evolutionary trajectory toward more complex resistance profiles [26].

From a clinical governance and surveillance perspective, this pattern has important implications. The predominance of first-line resistance markers, alongside relatively limited second-line resistance, suggests a critical window for intervention, in which strengthened diagnostic surveillance, early detection of isoniazid and rifampicin resistance, and rapid optimization of treatment regimens could prevent further escalation toward fluoroquinolone and injectable resistance. Monitoring mutation proxies across key resistance loci, therefore, provides not only molecular epidemiological insight but also a governance-relevant early signal of resistance evolution, enabling health systems to prioritize facilities or patient populations that require intensified diagnostic oversight and treatment stewardship. These findings highlight how programmatic changes in treatment policy can shape resistance landscapes, reinforcing the importance of integrating clinical, laboratory, and policy perspectives when interpreting molecular surveillance data [27,28].

### 4.3. Temporal Patterns in Drug-Resistance Prevalence

Interpretation of temporal variation in resistance-associated mutation proxies should consider concurrent changes in DR-TB treatment regimens in South Africa during the study period (2021–2024). National and global guidelines [29] have increasingly shifted toward all-oral treatment regimens, with reduced use of second-line injectable agents such as amikacin and kanamycin. This transition alters selective drug pressure within the population. It may contribute to the observed decline in second-line resistance-associated mutation proxies (e.g., *gyr*A and *rrs*) and to shifts in the relative contribution of first-line resistance markers, such as *inh*A. At the same time, persistent detection of second-line resistance proxies likely reflects legacy resistance, ongoing transmission of resistant strains, or delayed programmatic response, underscoring the continued relevance of these markers for surveillance and clinical governance.

Marked temporal variation in the prevalence of resistance-associated mutation proxies observed in this study is consistent with reports from other molecular epidemiology studies showing that drug-resistance mutations in *Mycobacterium tuberculosis* do not emerge uniformly over time but fluctuate according to transmission dynamics, treatment pressures, and diagnostic practices. In our dataset, distinct temporal peaks were observed across resistance loci: *gyr*A and *rrs* mutation proxies peaked in 2023, and *inh*A increased later in 2024, indicating shifting resistance profiles across drug classes. Similar heterogeneity in mutation distribution has been described in other studies, where resistance-associated mutations vary over time and across populations as selective pressure from treatment regimens changes. These findings support the concept that drug resistance evolves dynamically and is influenced by both programmatic and epidemiological factors, rather than following a single linear trend [30]. In our dataset, sustained baseline detection of resistance markers punctuated by short-term peaks suggests episodic amplification of resistant strains or localized transmission events. Such temporal fluctuations underscore the importance of longitudinal surveillance using routinely generated molecular diagnostic data, as short-term increases in specific mutation proxies may signal emerging resistance patterns that require programmatic review and clinical governance intervention. Annual aggregation showed a decline in overall mutation proxy prevalence between 2022 and 2024, although this trend should be interpreted cautiously. The early years of the study coincided with residual disruptions from the COVID-19 pandemic, which reduced TB service access and diagnostic activity in many settings, often limiting testing to patients with severe disease or high clinical suspicion. As TB services recovered and diagnostic capacity expanded in subsequent years, testing likely included a broader and more representative clinical population. Consequently, the observed decline in proportional prevalence may reflect changes in testing patterns rather than a true reduction in drug-resistant tuberculosis, despite an increase in the absolute number of detected cases.

### 4.4. Age-Stratified Distribution of Drug-Resistance Mutation Proxies

Resistance-associated mutation proxies were detected across all adult age groups, with notable variation in both prevalence and resistance profiles. A higher burden of resistance-associated mutations among adults has important implications for TB management, as this age group represents the most socially and economically active segment of the population and therefore plays a key role in ongoing transmission of drug-resistant *Mycobacterium tuberculosis*. Adults are also more likely to have prior TB treatment exposure, treatment interruption, or delayed diagnosis, factors that contribute to the development and amplification of drug resistance. Consequently, the predominance of resistance markers in adults highlights the need for rapid molecular resistance testing, prompt initiation of appropriate second-line regimens, and strengthened adherence support to prevent further transmission and progression to more complex resistance patterns [31,32].

Higher mutation-proxy prevalence in adults (especially in economically active ages) usually reflects where TB transmission and diagnosis concentrate: adults have higher social mixing and workplace/community exposure, and they also account for the bulk of TB case detection, so resistance markers are more often observed and have greater programmatic impact. In African datasets, *katG*/*inhA* patterns also differ by age, with *katG* frequently predominating across the economically active group, reinforcing the need for age-targeted adherence support and contact-tracing intensity where resistant TB circulates [21]. Our findings of very high prevalence in 15–24 years, dominated by *katG* with minimal second-line resistance, are more consistent with ongoing transmission of isoniazid-resistant strains than with stepwise acquisition during repeated treatment episodes. This interpretation is biologically plausible because *katG* Ser315Thr is a high-frequency, “low fitness cost” mutation that can be transmitted efficiently [33] and is also widely reported as the dominant INH-resistance mutation in African and global summaries [23].

Adolescents and young adults represent a highly mobile population, and the concentration of *katG*-associated resistance in this group is consistent with ongoing community-level spread of first-line drug-resistant strains [21,34]. Importantly, the relative absence of second-line resistance proxies indicates that fluoroquinolones and injectable agents remain largely preserved, highlighting a critical window for early intervention. In contrast, a laboratory-based surveillance study on second-line resistance among MDR patients in Ethiopia revealed that although the majority of MDR isolates included in the study were susceptible to second-line treatment (94.02%), a small proportion (6%) of the isolates were resistant to at least one of the second-line drugs, most likely among new TB cases. The study further reported the occurrence as acquired resistance due to treatment failure, leading to late resistance emergence [35]. These findings underscore the importance of routine upfront molecular drug-susceptibility testing in younger patients to ensure timely regimen optimization and prevent resistance amplification. From a programmatic perspective, age-stratified molecular surveillance can serve as an early warning signal, enabling targeted prevention, intensified case-finding, and youth-focused adherence and engagement strategies to interrupt transmission before progression to more complex, drug-resistant tuberculosis patterns. In contrast, the 25–34- and 35–44-year age groups contributed the largest absolute numbers of mutation-positive tests and exhibited the greatest diversity of resistance patterns, including notable detection of *gyrA* and *rrs.* These age groups comprise the most socioeconomically active part of the population. Individuals aged 25 to 44 have higher mobility, greater occupational exposure, and larger social networks, all of which facilitate sustained community transmission. The high prevalence of mutation-positive tuberculosis in this demographic shows that transmission occurs among economically productive, socially connected groups, perhaps maintaining transmission chains at the community level. Moreover, the diversity of resistance patterns to fluoroquinolones and second-line injectables may suggest the distribution of strains with more complex resistance profiles. This may be attributed to prior incomplete or inadequate treatment, transmission of already drug-resistant strains, and ongoing selective pressure from ongoing fluoroquinolone use for the treatment of other infections. Furthermore, the concentration of various resistance mutations in young-to-middle adulthood raises concerns about the long-term transmission of pre-XDR strains among highly interacting population groupings. If these people are not recognized or treated properly, they may become reservoirs for resistant tuberculosis, increasing the likelihood of future progression to XDR-TB. Among individuals aged ≥55 years, resistance remained dominated by *katG* mutations, with lower *prevalence of inhA mutations* but persistent detection of second-line resistance proxies. These findings are in collaboration with a study by Charan et al., which documented the most common mutation in INH monoresistance as *katG* (65.1%) and a lower prevalence of *inh*A resistance (28.1%) [36]. Conversely, a study on age-independent resistance in India reported lower mutation frequencies among older patients, suggesting that this may be attributed to the reactivation of older, drug-susceptible infections [37]. No resistance-associated mutation proxies were identified among the small number of tests from individuals under 15 years of age. Collectively, these findings indicate that drug-resistant TB affects adults across the lifespan, with peak burden and complexity concentrated in economically active age groups, consistent with cumulative exposure and prior treatment history. Comparable resistance profiles have been described in studies conducted in South Africa, Nigeria, Iran, and Thailand [38,39,40,41]. In contrast, a study focusing on the rural context of the TB burden reported the highest peak of TB incidence in those aged >70, suggesting causality to be associated with chronic disease, subsequently predisposing old age to TB [42].

### 4.5. Facility-Level Heterogeneity in Drug-Resistance Burden

Observed heterogeneity across facilities should be interpreted in the context of variable diagnostic volumes, as small sample sizes may amplify apparent differences and limit generalizability. Substantial heterogeneity in resistance burden was observed across health facilities, indicating that drug-resistant *Mycobacterium tuberculosis* is unevenly distributed, with certain facilities likely functioning as focal points of sustained transmission and resistance amplification rather than reflecting a uniform district-wide phenomenon. Such variability is consistent with differences in local transmission dynamics, patient case mix, referral patterns, and service delivery factors, and highlights the limitations of relying on aggregated program-level indicators to guide treatment decisions [40,43].

Clinically, patients presenting in high-burden facilities face an increased risk of initial regimen mismatch if standardized approaches are applied without timely molecular drug-susceptibility testing, potentially leading to early treatment failure and prolonged infectiousness [43]. Within a facility-level governance and early-warning framework, this heterogeneity becomes actionable intelligence: routine aggregation of molecular resistance signals enables early identification of facilities requiring intensified diagnostic coverage, clinical oversight, adherence support, and infection prevention measures, thereby supporting anticipatory, targeted interventions to contain resistance before progression to more complex drug-resistant TB patterns. Several sites demonstrated extremely high mutation proxy prevalence, including facilities with 100% positivity among tested samples, although small denominators strongly influence such estimates. In contrast, F4 contributed the largest absolute number of mutation-positive records, reflecting its high diagnostic throughput rather than solely its disproportionately high prevalence. These findings underscore the importance of interpreting facility-level resistance prevalence in conjunction with diagnostic volume to avoid overestimating resistance burden in low-volume facilities or underestimating the contribution of high-volume referral centers.

### 4.6. Persistence of Resistance Detection over Time

The prolonged persistence of resistance-associated mutation detection at selected facilities, most notably F2 and F4, indicates that drug-resistant *Mycobacterium tuberculosis* is being encountered continuously within these catchment areas rather than arising from sporadic or isolated cases. Sustained mutation positivity over periods of up to 15 months suggests ongoing transmission, recurrent presentation of resistant disease, or persistent programmatic gaps, such as delayed diagnosis, suboptimal regimen matching, or challenges with treatment adherence and continuity of care [33]. The particularly prolonged recurrence of fluoroquinolone-associated *gyrA* mutation proxies at F4, extending over 27 months with uninterrupted runs of up to 14 months, is of great clinical and programmatic concern, as it signals sustained second-line resistance pressure in a high-burden setting. From a treatment perspective, this threatens the effectiveness of cornerstone MDR-TB regimens and underscores the need for early, routine molecular drug-susceptibility testing and heightened clinical oversight at affected facilities [44]. Within a facility-level governance and early-warning framework, such persistence should be interpreted as a red-flag indicator warranting prioritized intervention, including intensified diagnostic coverage, regimen review, adherence support, and infection prevention measures, to prevent further amplification and entrenchment of second-line drug-resistant tuberculosis.

### 4.7. Mutation Persistence as a Proxy Indicator of Clinical Governance Stress

Conventional clinical governance frameworks in TB programs are largely reactive, responding to treatment failure, poor adherence, or established drug resistance after adverse outcomes have already occurred [45]. Findings from this study support an alternative, predictive framing in which routinely generated molecular diagnostic data are repurposed as early-warning indicators of governance stress from a clinical perspective at the facility level. The monitoring and use of drug-resistance trends could be critical in ensuring evidence-based decision-making at individual facilities to minimize the risks of treatment failure, poor clinical outcomes, and possible progression to severe forms of drug resistance.

Facility-level persistence analyses revealed prolonged, uninterrupted periods of proxy detection for resistance-associated mutations at selected sites, with continuous runs lasting up to 15 months. Sustained mutation positivity over time, particularly when observed across multiple resistance-associated loci, likely reflects more than ongoing transmission or complex case mix alone. Instead, such patterns plausibly indicate weaknesses in upstream clinical governance processes, including delayed regimen optimization, inconsistent clinical guideline review and updating, suboptimal adherence support, or gaps in treatment outcome monitoring.

Within this framework, mutation persistence is conceptualized not solely as a microbiological phenomenon but also as a proxy indicator of strains, potential strains, and possible weaknesses in the governance of healthcare services. We propose operationalizing a Mutation Persistence Threshold as the detection of one or more resistance-associated mutation proxies for six or more consecutive months at a facility. Exceeding this threshold signals accumulated, unmet health services governance and oversight needs and should prompt structured interventions to address community and facility factors to address the emerging trend.

Clinical governance responses triggered by sustained persistence may include targeted clinical audits of drug-resistant TB cases, intensified adherence support and patient tracing, and multidisciplinary regimen review, particularly in facilities demonstrating persistent fluoroquinolone-associated mutation proxies. Importantly, this approach requires no additional laboratory testing, novel diagnostics, or complex modeling. Instead, it transforms existing routine molecular outputs into actionable clinical governance intelligence to improve the quality of TB services.

By shifting clinical governance from retrospective oversight to anticipatory intervention, persistence-based metrics enable earlier identification of facilities at risk of sustained drug-resistant TB burden. This predictive framing aligns with data-informed health-system strengthening priorities. It provides a practical pathway for integrating laboratory surveillance into routine care and for improving workflow in TB in high-burden, resource-constrained settings. These constructs should be interpreted as conceptual frameworks intended to support interpretation of routine data patterns, rather than as validated indicators of system performance. Despite these limitations, mutation proxies remain programmatically valuable as rapid, routinely available indicators that can support population-level surveillance and early warning systems in resource-constrained settings. Future studies incorporating inferential statistics and longitudinal modeling would further strengthen the interpretation of temporal trends observed in this study.

### 4.8. Facility-Level Governance Triage Using a Governance Priority Score

Most analyses of drug-resistant TB focus on identifying where resistance prevalence is highest. While epidemiologically informative, such descriptions offer limited guidance on how to prioritize scarce health system resources across heterogeneous facilities to improve TB care. In this study, we adopt a systems-oriented inversion, reframing the analytic question from where resistance is high to where clinical governance intervention is most urgently required.

To support this reframing, we propose a Facility-Level Governance Priority Score (GPS) that integrates four dimensions derived entirely from routine molecular diagnostic data: (i) facility-level prevalence of resistance-associated mutation proxies; (ii) mutation diversity across key resistance loci (*katG*, *inhA*, *gyrA*, *rrs)*; (iii) persistence of mutation detection over consecutive months; and (iv) diagnostic volume. The proposed GPS should be interpreted as a conceptual, hypothesis-generating framework derived from observed data patterns rather than a validated predictive tool, and should be further evaluated across diverse settings.

This composite perspective recognizes that facilities with moderate prevalence, but prolonged persistence or high diagnostic throughput, may contribute disproportionately to the ongoing drug-resistant TB burden. Conversely, facilities with very high prevalence estimates derived from small test numbers may warrant closer monitoring rather than immediate intensive intervention. The GPS, therefore, moves beyond prevalence alone, incorporating temporal continuity, resistance complexity, and service pressure. Using this framework, facilities can be conceptually stratified into three clinical governance priority categories: governance-critical facilities exhibiting convergence of high prevalence, multi-locus diversity, prolonged persistence, and substantial volume; governance-watch facilities demonstrating intermediate or emerging burden; and governance-stable facilities characterized by low prevalence, limited diversity, and minimal persistence. The GPS is not intended as a rigid scoring algorithm but as a pragmatic decision-support tool to guide proportional clinical governance responses, including clinical audits, feedback to patient-facing clinicians, adherence interventions, review of clinical guidelines and regimens, and supervisory support. By translating routine molecular surveillance outputs into a structured clinical governance triage model, this approach strengthens the linkage between laboratory data and health-system optimization, enabling more efficient and anticipatory responses to drug-resistant TB.

### 4.9. Facility-Level Governance Triage Model Based on Routine Molecular Diagnostic Data

In current practice, molecular diagnostic data are predominantly used as patient-level clinical tools, with governance actions typically triggered only after treatment failure, deteriorating program indicators, or recognized outbreaks. This downstream, reactive configuration limits health systems’ capacity to respond proactively to emerging patterns of drug-resistant TB. The proposed Facility-Level Governance Triage Model reorients the use of routine diagnostic data toward anticipatory, system-level intelligence. By aggregating mutation proxy data at the facility level, resistance signals can cluster and acquire operational meaning beyond individual patient management. Rather than focusing exclusively on prevalence, the model identifies facilities where clinical governance intervention is most urgently required. Four routinely available indicators, mutation proxy prevalence, mutation diversity, mutation persistence, and diagnostic volume, are jointly interpreted to provide a multidimensional assessment of resistance burden, complexity, temporal continuity, and service demand. Individually, each indicator offers limited insight; collectively, they inform prioritization of governance intensity. These indicators are synthesized within the GPS framework to stratify facilities into governance-priority, governance-watch, and governance-stable categories, each linked to proportionate governance actions ranging from targeted audits and diagnostic and treatment regimen reviews to enhanced surveillance and clinical outcome monitoring. This classification is exploratory and intended to illustrate how routine data may be structured to support prioritization, rather than to define validated operational categories. The model thus reframes facility-level heterogeneity from a descriptive epidemiological observation into an operational governance tool.

### 4.10. Temporal Lag Between Resistance Detection and Clinical Governance Response

A recurring challenge in the performance of TB programs is the temporal disconnect between early resistance detection and an appropriate clinical governance response to tailor diagnostic and treatment approaches. While molecular diagnostics enable rapid identification of resistance-associated mutations, governance actions such as intensified supervision or program-level intervention are often initiated only after sustained adverse outcomes become apparent. This suggests that health system governance systems may lack an effective and responsive mechanism for measuring, monitoring, and responding to early warning signals of gaps and weaknesses in program performance.

Building on persistence analyses, we introduce the concept of a Clinical Governance Lag Index, defined as the interval between the initial detection of resistance-associated mutation proxies, their persistence over time, and the eventual escalation to prompt an appropriate clinical governance response. In this framework, prolonged uninterrupted runs of mutation-positive months represent accumulated, unmet governance signals rather than isolated microbiological events.

Facilities demonstrating continuous mutation detection for 12–15 months may therefore be conceptualized as governance-lag hotspots, where early resistance signals were repeatedly detected but not translated into timely intervention. In such settings, persistence likely reflects both ongoing transmission and a complex case mix, as well as a delayed or insufficient clinical governance response.

Recognizing governance lag as a system-level phenomenon reframes mutation persistence as an indicator of temporal misalignment between surveillance and action. Embedding predefined persistence thresholds into routine program oversight could strengthen governance memory, reduce delays in intervention, and shorten the duration during which drug-resistant TB remains unaddressed at the facility level. This temporal perspective reinforces the role of routine molecular surveillance as an early-warning system and supports more responsive, anticipatory clinical governance.

### 4.11. Strengths and Limitations

#### 4.11.1. Strengths

This study leverages a large, multi-year dataset of routinely generated molecular diagnostic records to capture real-world patterns of drug resistance across multiple health facilities in a high-burden district. Because mutation proxy data were generated as part of standard clinical care, the findings are highly relevant to routine programmatic decision-making and reflect resistance patterns encountered in everyday practice rather than those observed in selected research cohorts. The inclusion of age-stratified analyses adds epidemiological depth, allowing for assessment of how resistance burden and complexity vary across the adult lifespan and highlighting the population groups that contribute most to the observed resistance patterns.

#### 4.11.2. Limitations

Several limitations should be considered when interpreting the findings of this study. The analysis was limited to mutations detectable by routine molecular diagnostic assays and did not include resistance-associated genes such as pncA, embB, Rv0678, ddn, and others that require whole-genome sequencing or specialized assays. As such, the findings reflect programmatically detectable resistance patterns rather than the full molecular spectrum of drug resistance.

First, resistance was inferred using assay-derived mutation proxies rather than sequencing-confirmed mutations. These proxies reflect genotypic resistance within predefined genomic regions targeted by routine molecular diagnostic platforms and may not capture the full spectrum of resistance mechanisms or distinguish between specific genetic variants.

Second, the analysis was limited to mutations detectable by routine diagnostic assays, which primarily target genes such as *rpoB*, *katG*, *inhA*, and *gyrA*. As a result, resistance-associated mutations in other genes, including pncA, embB, *rrs*, Rv0678, rrl, and others, were not assessed. The findings should therefore be interpreted as representing programmatically detectable resistance patterns, rather than comprehensive molecular resistance profiling.

Third, molecular diagnostics identify genotypic resistance, which may not always correspond directly to phenotypic DST. Some level of discordance between genotypic and phenotypic resistance is well documented, particularly for mutations associated with low-level or borderline resistance. This discordance reflects inherent differences in detection principles between molecular assays and culture-based methods. In this study, mutation proxies are therefore interpreted as assay-derived molecular indicators of resistance-associated genetic regions, rather than definitive measures of phenotypic resistance. Accordingly, the findings should be interpreted as indicative of genotypic resistance patterns for surveillance and programmatic purposes, rather than as confirmation of phenotypic drug resistance. Despite this limitation, molecular diagnostics remain the cornerstone of rapid TB resistance detection and are widely endorsed for surveillance in high-burden settings.

Fourth, diagnostic practices and testing volume evolved over the study period as molecular testing was progressively scaled up. This expansion likely influenced the observed temporal trends and may limit direct comparability across years.

Fifth, the study was based on routine surveillance data, which may be subject to variability in data completeness, testing practices, and facility-level reporting. These factors may have influenced observed patterns in resistance prevalence and persistence.

Sixth, the study was not designed to provide statistically powered inferential analysis, and therefore does not support causal inference or generalizable conclusions. Instead, the findings represent descriptive patterns within a real-world programmatic dataset, intended to identify operationally relevant trends.

Finally, the governance framework proposed in this study has not been externally validated and should be interpreted as a conceptual model. Further research is required to assess its predictive validity, feasibility, and impact on clinical and programmatic outcomes.

### 4.12. Recommendations

The findings of this study suggest that routinely generated molecular diagnostic data may offer practical value for strengthening tuberculosis (TB) program monitoring and governance. In particular, incorporating routine assessment of resistance-associated mutation proxies into existing surveillance systems could support earlier identification of emerging resistance patterns. Beyond prevalence-based monitoring, the results indicate that persistent detection of mutation proxies across consecutive time periods may serve as a useful programmatic signal. Where feasible, health systems may consider defining operational thresholds for persistence (e.g., detection over multiple consecutive months) to guide further investigation. Such signals could prompt targeted actions, including focused clinical audits, review of treatment practices, and strengthened adherence support, depending on local context and available resources. The observed facility-level heterogeneity also highlights the importance of strengthening linkages between laboratory surveillance and program-level decision-making to ensure that routinely generated data are effectively translated into timely and context-sensitive responses. While the proposed governance prioritization framework (GPS) provides a structured approach for organizing resistance-related indicators, it should be interpreted as a conceptual and exploratory tool. Further research is required to assess its feasibility, generalizability, and potential impact in different high-burden settings. Overall, these findings support the need for integrated, data-informed approaches that combine molecular surveillance with clinical and programmatic data to improve the monitoring and management of drug-resistant TB.

## 5. Conclusions

In conclusion, this study demonstrates that routinely generated molecular diagnostic data contain valuable, yet underutilized, signals for understanding patterns of drug-resistant *Mycobacterium tuberculosis* within routine program settings. A substantial and heterogeneous burden of resistance-associated mutation proxies was identified across facilities in KSDLM, with isoniazid resistance predominating alongside temporal variation and facility-level differences. Importantly, the identification of mutation persistence and clustering highlights actionable patterns that extend beyond descriptive reporting. These findings underscore the potential of integrating molecular surveillance data into clinical governance to support more targeted, responsive, and context-specific tuberculosis control strategies in high-burden settings.

The persistent detection of mutation proxies at selected facilities suggests the potential for sustained transmission dynamics or ongoing treatment-related selective pressure. These patterns, while exploratory, indicate that routine molecular surveillance data may provide additional insights beyond individual patient management, particularly when examined longitudinally and across service delivery contexts. The proposed Facility-Level Governance Prioritization Score (GPS) is presented as a conceptual, exploratory framework for organizing routine molecular data within a structured, facility-level perspective. While it demonstrated the ability to differentiate patterns across facilities within this dataset, it should not be interpreted as a validated decision-making tool. Further research is required to assess its feasibility, reproducibility, and applicability in other settings. Overall, the findings support the potential value of integrating routine molecular surveillance data with broader clinical and programmatic information to improve the monitoring of drug-resistant TB. Importantly, the study demonstrates that routinely generated molecular diagnostic data can be leveraged not only to quantify resistance but also to identify patterns of persistence and facility-level clustering. These insights provide actionable signals for strengthening clinical governance, enabling more responsive, targeted, and context-specific TB control strategies. However, given the descriptive nature of the analysis and data limitations, these insights should be interpreted cautiously and viewed as a foundation for future, more robust investigations.

## Figures and Tables

**Table 1:** Overall prevalence of resistance-associated mutation proxies.

Resistance-Associated Locus	Mutation Proxy Detected, n (%)	Total Tests
Any mutation proxy	1007 (72.7)	1386
*katG*	724 (52.2)	1386
*inhA*	280 (20.2)	1386
*gyrA* (any region)	149 (10.8)	1386
*Rrs*	125 (9.0)	1386
*Eis*	0 (0.0)	1386

**Table 2:** Age-stratified prevalence of resistance-associated mutation proxies.

Age Group (Years)	Tests, n	Any Mutation n (%)	*katG* n (%)	*inhA* n (%)	*gyrA* n (%)	*rrs* n (%)
<15	4	0 (0.0)	0	0	0	0
15–24	157	153 (97.5)	129 (82.2)	24 (15.3)	1 (0.6)	0 (0.0)
25–34	322	231 (71.7)	128 (39.8)	60 (18.6)	89 (27.6)	34 (10.6)
35–44	389	265 (68.1)	155 (39.8)	134 (34.4)	17 (4.4)	46 (11.8)
45–54	244	149 (61.1)	103 (42.2)	46 (18.9)	0 (0.0)	3 (1.2)
≥55	270	209 (77.4)	209 (77.4)	16 (5.9)	42 (15.6)	42 (15.6)

**Table 3:** Governance triage classification of selected high-impact facilities (F1–F5).

Facility Code	Diagnostic Volume (n)	Any Mutation Proxy n (%)	Key Resistance Features	Persistence/Volume Context	Governance Triage Category	Rationale for Classification
F1	16	16 (100%)	Predominantly first-line resistance; no *gyrA* or *rrs* detected	Very low volume; no documented persistence	Governance-watch	Extremely high proportional prevalence based on small numbers; requires monitoring rather than immediate intensive intervention.
F2	13	13 (100%)	High second-line resistance burden (*gyrA* 69.2%, *rrs* 46.2%)	Low volume but high resistance complexity	Governance-critical	Extensive fluoroquinolone and injectable resistance signals indicate elevated governance risk.
F3	75	71 (94.7%)	Resistance is largely limited to first-line drugs	Moderate volume; referral-level case mix	Governance-watch	High prevalence expected in a tertiary referral setting; patterns reflect case complexity.
F4	699	589 (84.3%)	Significant second-line resistance (*gyrA* 17.5%, *rrs* 11.7%)	Very high volume; prolonged persistence (up to 15 months)	Governance-critical	Convergence of high absolute burden, resistance diversity, and persistence indicates sustained governance stress.
F5	13	11 (84.6%)	Prominent fluoroquinolone resistance (*gyrA* 46.2%)	Low–moderate volume; no *RRs* detected	Governance-watch	High proportional resistance to the second-line signal warrants enhanced surveillance.

## Data Availability

The datasets generated and/or analyzed during this study are not publicly available due to data protection regulations and the Eastern Cape Department of Health’s governance policies. However, de-identified and aggregated data may be made available from the corresponding author upon reasonable request, subject to approval from the relevant data custodians and institutional authorities.

## Abbreviations

The following abbreviations are used in this manuscript:

F	Facility
GPS	Governance Priority Score
KSD	King Sabata Dalindyebo
KSDLM	King Sabata Dalindyebo Local Municipality
MDR	Multidrug resistant
O.R	Oliver Reginald
TB	Tuberculosis
XDR	Extensively drug resistant
